# Using gene co-expression network analysis to predict biomarkers for chronic lymphocytic leukemia

**DOI:** 10.1186/1471-2105-11-S9-S5

**Published:** 2010-10-28

**Authors:** Jie Zhang, Yang Xiang, Liya Ding, Kristin Keen-Circle, Tara B Borlawsky, Hatice Gulcin Ozer, Ruoming Jin, Philip Payne, Kun Huang

**Affiliations:** 1 Department of Biomedical Informatics, The Ohio State University, OH, USA; 2 Comprehensive Cancer Center, BISR, The Ohio State University, OH, USA; 3 Nationalwide Children’s Hospital, OH, USA; 4 Center for Clinical and Translational Science, The Ohio State University, OH, USA; 5 Department of Computer Science, Kent State University, OH, USA

## Abstract

**Background:**

Chronic lymphocytic leukemia (CLL) is the most common adult leukemia. It is a highly heterogeneous disease, and can be divided roughly into indolent and progressive stages based on classic clinical markers. Immunoglobin heavy chain variable region (IgV_H_) mutational status was found to be associated with patient survival outcome, and biomarkers linked to the IgV_H_ status has been a focus in the CLL prognosis research field. However, biomarkers highly correlated with IgV_H_ mutational status which can accurately predict the survival outcome are yet to be discovered.

**Results:**

In this paper, we investigate the use of gene co-expression network analysis to identify potential biomarkers for CLL. Specifically we focused on the co-expression network involving ZAP70, a well characterized biomarker for CLL. We selected 23 microarray datasets corresponding to multiple types of cancer from the Gene Expression Omnibus (GEO) and used the frequent network mining algorithm CODENSE to identify highly connected gene co-expression networks spanning the entire genome, then evaluated the genes in the co-expression network in which ZAP70 is involved. We then applied a set of feature selection methods to further select genes which are capable of predicting IgV_H_ mutation status from the ZAP70 co-expression network.

**Conclusions:**

We have identified a set of genes that are potential CLL prognostic biomarkers IL2RB, CD8A, CD247, LAG3 and KLRK1, which can predict CLL patient IgV_H_ mutational status with high accuracies. Their prognostic capabilities were cross-validated by applying these biomarker candidates to classify patients into different outcome groups using a CLL microarray datasets with clinical information.

## Background

Chronic lymphocytic leukemia (CLL), also called B-cell CLL, is the most common type of leukemia, which mainly affects adults. Nearly 100,000 Americans live with CLL, most of them over fifty years old. Rates of CLL incidence are increasing, and there is no known cure for the disease [1]. For patients diagnosed with CLL, staging or classification systems such as the widely adopted Rai and Binet staging systems can categorize the patients into classes with different risk levels [2]. However, currently these systems still have difficulty in discriminating indolent and progressive CLL. Specifically, some patients remain in the beginning or indolent stage of the disease and do not require treatment, which involves numerous undesirable side effect, for time periods of up to ten or more years [3,4]. In contrast, some patients experience very aggressive disease in a short time period, characterized by rapid white blood cell doubling time, and requiring immediate treatment. These differences delineate two distinct groups of patients: indolent and progressive CLL. Those with the non-progressive manifestation of the disease rarely need treatment until the disease transforms into an aggressive state and they become increasingly symptomatic [5]. Early determination of the CLL subtype is central to the goal of providing evidence-based adaptive therapies [6]. Such adaptive therapies can decrease disease-related mortality and increase quality of life. Several biomarkers have proven helpful in supporting such disease staging [4]. For example, the mutational status of IgV_H_ genes have been named in multiple studies as a biomarker for CLL disease progression [5,7,8]. However, testing IgV_H_ mutation status is costly and is not readily available in all clinical settings. Recently, cell membrane proteins such as ZAP70 (Zeta-chain-associated protein kinase 70) and CD38 have been proposed as biomarkers for CLL prognosis [5,9,10]. Positive ZAP70 or CD38 tests have been shown to correlate with progressive CLL. While the identification of ZAP70 and its prognostic value represents progress toward more widespread and accessible CLL staging, ZAP70 testing only yields definitive results when conducted during later, symptomatic phases of disease progression [11]. And CD38 was later found to be an independent biomarker [12]. A more desirable method would be to determine biomarkers or phenotypic parameters that are able to definitively determine the likelihood with which a patient may develop rapid disease progression early in the pathophysiologic development of CLL. Thus researchers are still searching for new CLL biomarkers as illustrated in recent reports on correlations between LAG3 and LPL level and the mutation status of IgV_H_ genes in CLL patients [13].

Given the preceding motivation to discover and utilize more timely, effective, and accessible CLL biomarkers, we have investigated the use of gene co-expression network analysis to identify such prognostic factors. Gene co-expression networks are established by connecting genes with similar expression profiles across a group of subjects or in multiple studies. The similarity of expression profiles is often measured by parameters such as the Pearson correlation coefficients (PCC, -1 ≤ PCC ≤ 1), with a PCC of 1 implying perfect correlation and PCC of -1 being completely negative correlation. In a recent study, by using the well-known breast cancer biomarkers BRCA1 and BRCA2 as anchor genes, the authors were able to discover a new breast cancer biomarker, HMMR, whose expression profile highly correlates with those of the two anchor genes [14].

**Figure 1:**
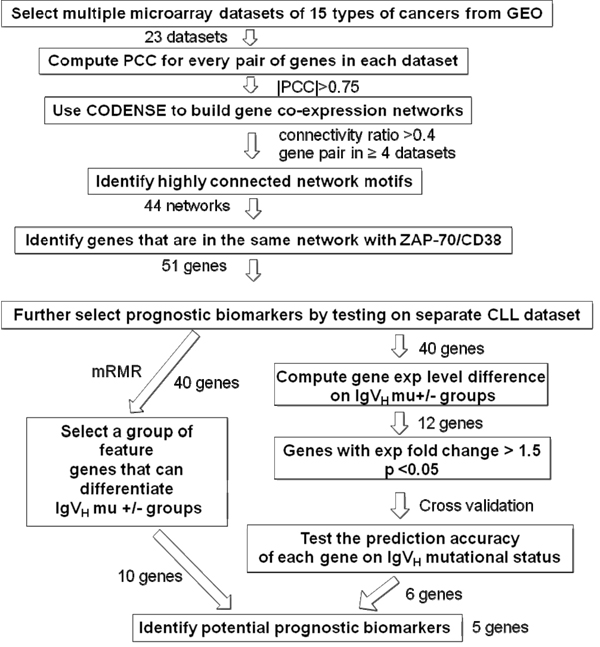
The workflow to identify genes co-expressed with ZAP70 in multiple cancer datasets using co-expression network analysis.

In this project, we took a similar approach by studying genes co-expressed with ZAP70 in multiple datasets. Specifically, we selected 23 microarray datasets corresponding to multiple types of cancers from the Gene Expression Omnibus (GEO) and used the CODENSE algorithm to identify highly connected gene co-expression network spanning the entire genome. We then narrowed down the gene list in the co-expression networks in which ZAP70 was involved by testing their capabilities for predicting the IgV_H_ status of the subjects using various machine learning feature selection methods. The workflow for this approach is summarized in Figure 1. The biomarkers identified from the workflow were then subjected to validation by testing their prognostic power on another CLL microarray dataset with patients clinical outcome information available.

## Results

### Identify genes in the co-expression network with ZAP70 using CODENSE

**Figure 2:**
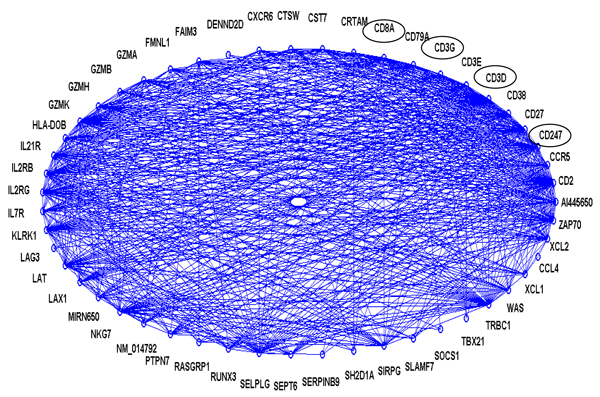
**The connectivity graph for Network 17.** The connectivity ratio *r* for this network is 0.4142. The names with circle are the genes which product known to interact with ZAP70.

**Figure 3:**
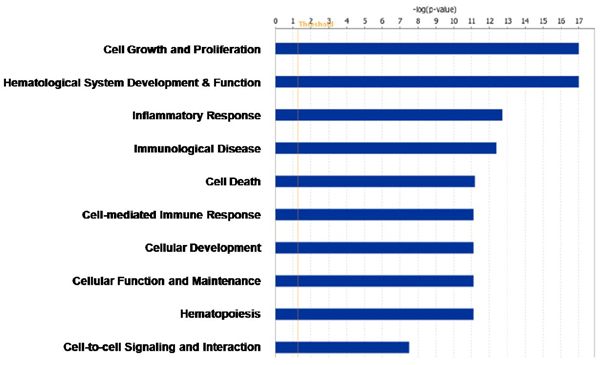
The top 10 enriched biological functions of Network 17 genes using IPA.

Using the CODENSE algorithm with the settings described in the Methods section, we identified 44 highly connected co-expression networks (connectivity ratio *r* > 0.4). Network 17 (shown in Figure 2, containing 51 genes) includes the well known CLL biomarker ZAP70. Interestingly, another CLL biomarker CD38 is also included in this network. Four genes CD8A, CD3G, CD247 and CD3D, whose products are known to interact with ZAP70, are also in this network. The GO-term enrichment analysis using IPA (Ingenuity Pathway Analysis) revealed highly enriched biological functions related to leukemia, such as cell growth and proliferation, hematological system development and function, inflammatory response, and immunological disease.(Figure 3).

### Identify genes in the ZAP70 co-expression network with differential expression levels between different IgV_H_ mutation groups in GDS1454 dataset

Among these 51 genes, we further selected genes whose expression levels can predict IgV_H_ mutation status using the three steps outlined in the Method section.

Table 1 summarizes the genes in Network 17 with p-values less than 0.05 and a mean expression fold change greater than 1.5 (except for ZAP70) between the IgV_H_ unmutated and mutated groups using Student’s t-tests. Out of the 51 genes in Network 17, 11 genes satisfied these criteria, with 10 genes up-regulated in the IgV_H_ unmutated group and one gene down-regulated in the same group. It is worth noting that although our selection criteria does not include the more conservative multiple t-test compensation methods such as Bonferroni test, out of the 12651 probesets in GDS1454, only 190 satisfied our criteria with 122 up-regulated and 68 down-regulated, which constitute a reasonable set of genes for further screening. In addition, in the GeneCards database, we identified 120 candidate genes, which products interact with ZAP70. Out of the 120, 9 genes’ expression profiles satisfied our selection criteria, with 4 of the 9 being included in Network 17 (the names with circle in Figure 2).

**Table 1:** Statistics of comparison between the IgVH unmutated and mutated groups for Network 17 genes.

Genes	p-values (Unmutated vs Mutated IgV_H_)	Mean fold change (Unmutated vs Mutated IgV_H_)	p-value (Patients vs Normal)
SH2D1A	1.3E-3	1.944	0.089
IL2RB	8.1E-5	1.821	4.8E-16
KLRK1	4.9E-3	1.813	0.0079
CD247	1.6E-4	1.807	7.1E-8
GZMB	3.1E-3	1.719	6.2E-11
CD3G	0.017	1.685	0.41
CD3D	1.4E-4	1.621	4.3E-16
GZMK	0.022	1.586	9.2E-11
CD8A	9.9E-5	1.576	3.5E-9
NKG7	8.3E-4	1.560	1.3E-9
ZAP70	7.9E-4	-1.403	5.5E-12
LAG3	0.023	-1.598	0.028

The p-values are the results of Student’s t-test of comparing the IgVH mutated vs. unmutated group, as well as comparing the CLL patient vs. normal group.

### Predicting capability of individual genes on IgVH mutational status

The preceding comparisons suggest that the co-expression network we have discovered is enriched with genes that differentially expressed between the IgV_H_ unmutated group and IgV_H_ mutated group. In addition, since these genes are all related to ZAP70, we focused on selecting candidate biomarkers from those listed in Table 1. We tested the predictive capacity of those 12 genes (11 genes identified from the above approach plus ZAP70) relative to IgV_H_ mutational status using a linear classifier. For each gene, the test was carried out using a cross-validation with 20% holdout of the samples, repeated 100 times. The results of these analyses, including average accuracy, are shown in Table 2. For the three genes with the highest accuracy (IL2RB, ZAP70, CD8A), we further explored the possibility of using aggregate features by testing two feature combinations. For each aggregate feature, the same test was conducted with the same setting as done on individual genes. We also examined the sub-cellular locations of each individual gene. Interestingly, most of the genes code for membrane proteins, which is a potential advantage for candidate biomarkers, since they can facilitate detection via effective and fast procedures such as flow cytometry.

**Table 2:** Accuracy of predicting IgVH mutational status with individual / combined potential biomarkers.

Genes	Prediction Accuracy	Sub-cellular location
SH2D1A	57.32%	cytoplasmic
**IL2RB**	68.84%	membrane
KLRK1	63.67%	membrane
**CD247**	66.03%	membrane
GZMB	57.13%	secreted
CD3G	62.52%	membrane
CD3D	64.27%	membrane
GZMK	57.58%	secreted
**CD8A**	68.31%	membrane
NKG7	64.94%	membrane
**ZAP70**	68.46%	cytoplasmic
LAG3	59.53%	membrane
**ZAP70+IL2RB**	73.22%	-
**ZAP70+IL2RB+CD8A**	74.62%	-

The names with bold letter indicate predicting accuracy above 65%. A linear classifier was used. The cross-validation was carried out with 20% holdout. Each test was carried out independently.

### Selecting gene features using mRMR

As indicated in the method section, we also used the mRMR webtool (http://penglab.janelia.org/proj/mRMR/) to select genes which form a broad feature set to differentiate the two groups. Specifically, we input the expression values of all the 51 genes and selected the setting with mutual information difference scheme based upon ten features (Table 3).

**Table 3:** The top ten genes selected by mRMR ordered by the mRMR score.

Order	Name	mRMR Score
1	IL2RB	0.101
2	LAG3	0.020
3	RASGRP1	0.029
4	CD8A	0.021
5	XCL1	0.011
6	ZAP70	0.018
7	CD79A	0.001
8	FMNL1	0.000
9	KLRK1	0.000
10	CST7	0.002

Table 2 and Table 3 have five genes in common: IL2RB, LAG3, CD8A, KLRK1 and ZAP70. Furthermore, as shown in Table2, IL2RB, CD8A and ZAP70 all show relatively high predictive capacity for IgV_H_ status, which suggests that IL2RB and CD8A are potential prognostic biomarkers besides ZAP70. In addition, CD247, KLRK1 and LAG3 are also good candidate biomarkers for CLL prognosis due to their high predicting accuracy as well as their representing distinct features between different IgV_H_ mutational groups. LAG3 has recently been identified as a potential CLL prognostic biomarker in another experimental study[13].

### Validate the prognostic capability of the identified biomarkers using new CLL microarray dataset (GSE10138)

**Figure 4:**
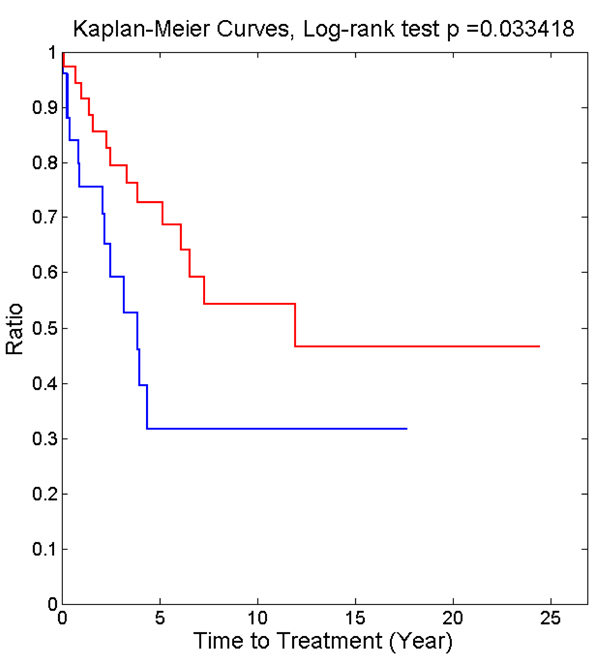
**The Kaplan-Meier curves of the two groups of CLL patients in the dataset GSE10138 using unsupervised K-mean clustering.** The biomarkers used to generate the survival curves are: ZAP70, LAG3, IL2RB, CD247, CD8A and KLRK1.

Figure 4 shows the Kaplan-Meier curves of patient time-to-treatment (TTT) from microarray data (GSE10138) of 61 CLL patients, using ZAP70 and all above identified biomarker candidates as features to categorize the patients into two risk groups. TTT is the time point when the disease evolves from indolent stage into progressive stage, signifying the switch from low to higher risk group, therefore is a suitable parameter to test our biomarkers for prognosis. The above biomarker candidates clearly separated the patients into two risk groups using the K-means algorithm (K=2), with the log-rank test p-value as low as 0.033. However, if only using ZAP70 as the feature to separate the patients, the difference of TTT between the two groups is not significant (log-rank test p > 0.05, figure not shown). Interestingly, with or without KLRK1, the patient grouping results and p-values stay the same.

## Discussion

**Figure 5:**
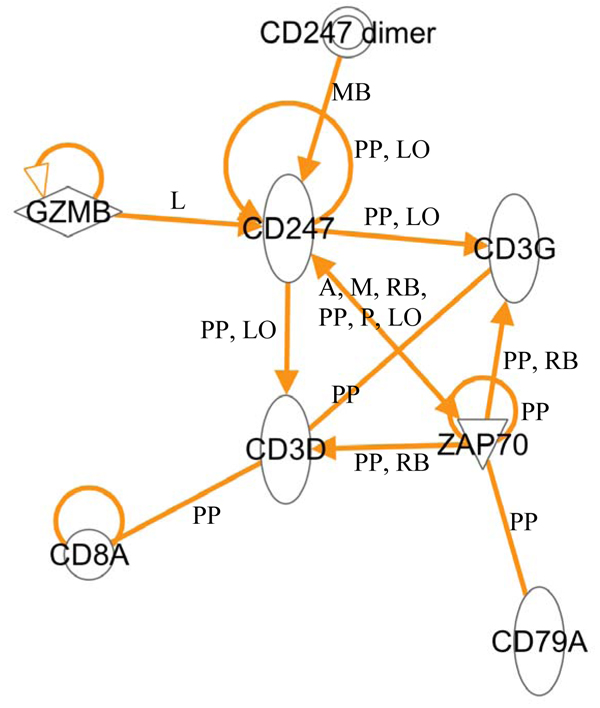
**The known interactions among potential prognostic biomarkers and ZAP70.** The interactions were extracted from Ingenuity Pathway Knowledge database. The abbreviations for interaction types: A: activation; L: proteolysis; M: biochemical modification; P: phosphorylation/dephosphorylation; LO: localization; MB: group/complex membership; PP: protein-protein binding; RB: regulation of binding.

As shown in Figure 5, except for KLRK1, all above biomarker candidates have been known to interact with each other and/or with ZAP70. Here we discuss the potential of the selected genes based on literature survey and our results:

***LAG3 (lymphocyte-activation gene 3)***: LAG3 product is involved in T-cell-dependent B-cell activation. It has been shown to be a potential biomarker using experimental methods in a recent study [13]. This observation not only partially validated our approach for identifying prognostic biomarkers for CLL, but also suggested that our method is able to identify even better biomarker, given that IL2RB and CD8A have stronger predictive power than LAG3.

***IL2RB (interleukin 2 receptor subunit beta)***: Expression of the IL2 receptor subunits IL2RB and IL2RG on B-cells has been known to be a sign of CLL [15,16]. Various drugs have been designed to target IL2 in CLL, even though it is not clear why some patients show relapse after the treatment [17]. However, currently we are not aware of any study relating IL2RB with IgV_H_ mutation status. Our results suggest that IL2RB has a great potential of being a prognostic biomarker for CLL.

***CD8A****and****CD247***: Both are T-cell surface antigens, but expression of CD8A on B-cells has been reported in CLL patients [18,19]. Since the samples for the data in GDS1454 are generated from mononuclear cells including both T-cells and B-cells, it is not clear what the origin of these molecules is. Regardless, they demonstrate comparable capacity in predicting IgV_H_ mutation status as ZAP70 and are worthy of further investigation.

***KLRK1 (killer cell lectin-like receptor superfamily K, member 1)***: KLRK1 is also called CD314. It is a member of C-type lectin-like family of type II cell surface glycoproteins, which is expressed by NK cells, CD8+ cells and certain types of T-cells [20]. KLRK1 is involved in transmitting activation signals into these types of cells, but it has never been associated with CLL or its prognosis. There is no known interaction between KLRK1 and other known or prognostic biomarkers identified in this paper, as indicated by its absence from the network generated using IPA based on known interactions (Figure 5). It is speculated that the expression level change in KLRK1 is probably a secondary effect of one or more of the rest of the biomarkers candidates, therefore whether including it or not seems not to affect the prognosis results.

In this paper, we employed the CODENSE algorithm to identify 44 gene co-expression networks using 23 cancer datasets. We found that the co-expression network containing ZAP70 is enriched with genes that show differential expression between the IgV_H_ unmutated and mutated groups, even though there is no CLL data included in the original 23 datasets from which the network was constructed. This finding suggests that the co-expression networks identified in this study can serve as a set of generic building blocks for biomarker selection and gene interaction in cancer studies[22].

A key issue in biomarkers discovery is to choose the candidates for experimental validation from vast amount of potential genes. Here we show that gene co-expression network analysis is an effective method for narrowing down the list of candidates. However, there are two limitations to this approach that should be noted: First, the effectiveness of this approach has not been determined by a prospective experimental study; and second, the approach is based on known biomarkers and may miss novel markers that involve in different mechanisms or regulation pathways. Therefore, currently we plan to expand our study on all the co-expression networks that have been identified using CODENSE. Another direction for the future study is to explore aggregate biomarkers of a combined group of gene products. We demonstrated the feasibility of this approach in Table2. However, a more rigorous and systematic screening for different combinations of genes is needed, which is part of our ongoing study.

## Conclusions

Using frequent gene co-expression analysis, we have identified a set of genes, IL2RB, CD8A, CD247, LAG3 and KLRK1, which are potential CLL prognostic biomarkers. Their prognostic capabilities were cross-validated by applying these biomarkers to classify patient survival groups using a CLL microarray datasets with patient clinical outcome information.

## Methods

### Data selection

We initiated this project by querying the GEO database using the term "chronic lymphocytic leukemia" [21]. Five GDS dataset results were returned from the query: GDS2676, GDS2643, GDS2501, GDS1454, and GDS1388. We filtered these results to identify datasets comparing patients in different groups; yielding the GDS1454 and GDS1388 data sets. GDS1454 is particularly important since it contains data obtained from the mononuclear cells of 111 subjects (11 normal subjects, 49 CLL patients without IgV_H_ mutation, and 51 CLL patients with IgV_H_ mutations). These GDS datasets were downloaded for analysis. In addition, a recently available CLL microarray dataset GSE10138 containing 68 patients was used to validate the biomarkers identified in the paper. Among them, the clinical information for 61 patients (33 with stable CLL and 28 with progressive disease) is available, and used in validation step.

### Co-Expression network discovery using CODENSE

As described earlier, we have previously used gene co-expression network analysis to identify novel biomarkers for breast cancer [22]. We applied a similar method in this project. In our approach, GEO was queried using terms "metastatic cancer”. Then only the datasets (GDS data) containing both normal and tumor tissues obtained from primary flash frozen biopsy (cell lines and secondary cultures were excluded) were selected. Using this method, 23 datasets from 15 types of cancer were selected. The Pearson correlation coefficients (PCC) for every pair of genes in every dataset were computed. Since we focus on gene pairs that are highly correlated, for each dataset we retained the gene pairs with |PCC| being 0.75 or higher.

The CODENSE algorithm was originally developed for identifying gene networks in multiple microarray datasets and is therefore suitable for our study [23]. We applied the CODENSE algorithm to the 23 lists of selected gene pairs as described above such that networks were constructed from gene pairs that appeared in at least 4 datasets. The networks with connectivity ratios *r* > 0.4 (i.e., given a co-expression network with *K* nodes and *L* edges, *r = L*/(*K*(*K-1*)/*2*)) were selected for further analysis.

### Test selected genes on a CLL dataset (GDS1454) using supervised methods

For the genes in the co-expression networks that included ZAP70, we further selected a subset of genes as potential prognosis markers for CLL by identifying genes whose expression levels can predict IgV_H_ mutation status. Out of the 51 genes from network 17, only 40 were present in the GDS1454 dataset, therefore only these 40 genes were examined in the following steps. Our approach to doing so includes three steps (also see Figure 1):

1. We compared their expression levels between the 49 patients without IgV_H_ mutation and the 51 patients with IgV_H_ mutations in GDS1454 and selected genes which demonstrated significant differential expression between the two groups.

2. The genes selected in step 1 were further tested for their capability of predicting IgV_H_ mutation status using a supervised linear classifier (as described in [24] and implemented in the *classify* function in Matlab which fit normal distributions to the groups) and a cross validation with 20% sample holdout, which is then repeated 100 times.

3. In addition to the tests on individual genes, we also applied a feature selection method, mRMR (minimum Redundancy Maximum Relevance), to select a group of feature genes from the gene list that can differentiate the two group patients. The mRMR was originally designed for gene selection in microarray data [25]. It allows us to select a subset of genes that can effectively distinguish the two groups of subjects (IgV_H_ unmutated vs. IgV_H_ mutated).

### Cross-validate the prognostic biomarkers with CLL dataset (GSE10138)

Unsupervised K-mean clustering (K=2) was performed 100 times (to ensure convergence and avoid local optimal results) on CLL microarray dataset GSE10138 using the expression levels of ZAP70, IL2RB, CD8A, CD247, LAG3 and KLRK1 as features. The dataset GSE10138 also contains the time-to-treatment (TTT) information for 61 patients, which is used to plot the Kaplan-Meier curves. Log-rank test was performed to determine the p-value of difference in TTT between the two patient groups.

### GO-term enrichment and pathway analysis using IPA

A commercially available pathway analysis package Ingenuity Pathway Analysis (IPA) was used to search the known interactions between identified biomarkers as well as to study the GO-term enrichment of the identified networks.

### Query other gene interaction database

To compare our results relative to ZAP70 gene co-expression with genes that are known interactants with ZAP70, we search for functional protein association in the GeneCards database (http://www.genecards.org/).

## Authors' contributions

JZ leads the project, selected most datasets, carried out the gene list analysis, and lead the manuscript writing. LD carried out the supervised learning and gene selection using mRMR. KKC identified the CLL datasets and carried out initial co-expression analysis for ZAP70. YX set up the codes for CODENSE and carried out the frequent co-expression network mining. TBB contributed to the development of the idea of the CLL biomarker discovery based on ZAP70 and CD38. HGO helped the initial processing of the CLL dataset. RJ supervised the tests of the network mining algorithms including CODENSE. PP first proposed the idea of searching for new CLL biomarkers and contributed to the writing of the manuscript. KH designed the whole workflow including the idea of using co-expression network for CLL biomarker discovery and contribute to the writing of the manuscript.

## Competing interests

The authors declare that they have no competing interests.
